# Supplementary material for: moSCminer: a cell subtype classification framework based on the attention neural network integrating the single-cell multi-omics dataset on the cloud
Source: PeerJ. 2024 Feb 26;12:e17006. doi: 10.7717/peerj.17006 (PMC10903350; doi:10.7717/peerj.17006)
Supplement: Supplemental Information 1 — Grid search was adopted for the model tuning, and the hyperparameters showing the best accuracy were selected. Row with the bolded font are the hyperparmeters selected. [file peerj-12-17006-s001.pdf]

Supplementary Table S1
Optimization results of the proposed method with the different combination of theparameters. Grid search was adopted for the model tuning, and the hyperparameters showing the best accuracy were selected. Row with the bolded font are the hyperparmeters selected.

GSE136718 Dataset

Size of the embedding vector	Size of the \bar{x}_i	# of nodes in FC layer	Dropout rate	Accuracy (train)	Accuracy (test)
128	64	100	0.6	1.000	0.976
128	64	100	0.6	0.938	0.976
128	64	100	0.6	1.000	0.952
128	64	100	0.6	1.000	0.976
128	64	100	0.6	1.000	0.976
128	64	100	0.4	0.938	0.952
128	64	100	0.4	1.000	0.976
128	64	100	0.4	1.000	0.976
128	64	100	0.4	1.000	0.976
128	64	100	0.4	1.000	0.976
128	64	100	0.2	1.000	0.952
128	64	100	0.2	1.000	0.976
128	64	100	0.2	1.000	0.952
128	64	100	0.2	1.000	0.976
**128**	**64**	**100**	**0.2**	**1.000**	**1.000**
128	64	100	0	0.938	0.976
128	64	100	0	0.938	0.952
128	64	100	0	1.000	0.905
128	64	100	0	1.000	0.952
128	64	100	0	0.938	0.976
128	64	50	0.6	0.875	0.905
128	64	50	0.6	1.000	0.952
128	64	50	0.6	0.875	0.952
128	64	50	0.6	0.938	0.952
128	64	50	0.6	1.000	0.976
128	64	50	0.4	0.875	0.976
128	64	50	0.4	0.813	0.952
128	64	50	0.4	1.000	0.976
128	64	50	0.4	1.000	0.976
128	64	50	0.4	0.875	0.976
128	64	50	0.2	1.000	0.976
128	64	50	0.2	1.000	1.000
128	64	50	0.2	0.938	0.976
128	64	50	0.2	1.000	0.976
128	64	50	0.2	0.750	0.952
128	64	50	0	1.000	0.929
128	64	50	0	1.000	0.881
128	64	50	0	1.000	0.976
128	64	50	0	1.000	0.952
128	64	50	0	1.000	1.000
256	128	100	0.6	1.000	0.976
256	128	100	0.6	0.938	1.000
256	128	100	0.6	1.000	1.000
256	128	100	0.6	0.938	0.976
256	128	100	0.6	0.875	0.976
256	128	100	0.4	0.938	1.000
256	128	100	0.4	1.000	0.976
256	128	100	0.4	0.938	1.000
256	128	100	0.4	1.000	0.976
256	128	100	0.4	1.000	1.000
256	128	100	0.2	1.000	1.000
256	128	100	0.2	1.000	1.000
256	128	100	0.2	1.000	1.000
256	128	100	0.2	1.000	1.000
256	128	100	0.2	1.000	0.976
256	128	100	0	1.000	0.929
256	128	100	0	0.875	0.976
256	128	100	0	0.938	0.952
256	128	100	0	1.000	0.952
256	128	100	0	0.875	1.000
256	128	50	0.6	1.000	0.976
256	128	50	0.6	0.813	0.976
256	128	50	0.6	0.813	0.976
256	128	50	0.6	0.813	0.952
256	128	50	0.6	1.000	0.976
256	128	50	0.4	1.000	0.976

256	128	50	0.4	1.000	0.976
256	128	50	0.4	1.000	0.952
256	128	50	0.4	1.000	0.976
256	128	50	0.4	1.000	0.976
256	128	50	0.2	1.000	1.000
256	128	50	0.2	1.000	0.976
256	128	50	0.2	1.000	1.000
256	128	50	0.2	1.000	1.000
256	128	50	0.2	1.000	0.976
256	128	50	0	1.000	1.000
256	128	50	0	1.000	0.929
256	128	50	0	0.875	0.976
256	128	50	0	0.875	0.952
256	128	50	0	1.000	1.000
64	32	100	0.6	1.000	0.905
64	32	100	0.6	1.000	0.905
64	32	100	0.6	0.875	0.929
64	32	100	0.6	0.875	0.929
64	32	100	0.6	0.750	0.929
64	32	100	0.4	0.938	0.929
64	32	100	0.4	0.875	0.952
64	32	100	0.4	1.000	0.952
64	32	100	0.4	0.875	0.952
64	32	100	0.4	0.938	0.976
64	32	100	0.2	1.000	1.000
64	32	100	0.2	0.875	0.952
64	32	100	0.2	1.000	0.952
64	32	100	0.2	1.000	0.952
64	32	100	0.2	1.000	0.952
64	32	100	0	0.938	0.952
64	32	100	0	1.000	0.905
64	32	100	0	1.000	0.952
64	32	100	0	1.000	0.952
64	32	100	0	1.000	0.976
64	32	50	0.6	0.938	0.929
64	32	50	0.6	0.688	0.929
64	32	50	0.6	0.938	0.952
64	32	50	0.6	0.938	0.881
64	32	50	0.6	0.875	0.905
64	32	50	0.4	0.938	0.952
64	32	50	0.4	0.938	0.976
64	32	50	0.4	0.813	0.976
64	32	50	0.4	1.000	0.976
64	32	50	0.4	1.000	0.929
64	32	50	0.2	0.938	0.952
64	32	50	0.2	0.938	0.976
64	32	50	0.2	0.938	0.976
64	32	50	0.2	1.000	0.976
64	32	50	0.2	1.000	0.976
64	32	50	0	1.000	0.786
64	32	50	0	1.000	0.952
64	32	50	0	1.000	0.810
64	32	50	0	1.000	0.976
64	32	50	0	1.000	0.786
64	32	50	0	1.000	0.905
64	32	50	0	1.000	0.976
64	32	50	0	0.938	0.881

GSE154762 Dataset

Size of the embedding vector	Size of the \bar{x}_i	# of nodes in FC layer	Dropout rate	Accuracy (train)	Accuracy (test)
128	64	100	0.6	0.864	0.922
128	64	100	0.6	0.864	0.906
128	64	100	0.6	0.864	0.911
128	64	100	0.6	0.909	0.922
128	64	100	0.6	0.909	0.906
128	64	100	0.4	0.955	0.911
128	64	100	0.4	0.909	0.933
128	64	100	0.4	0.955	0.933
128	64	100	0.4	1.000	0.928
128	64	100	0.4	1.000	0.917
**128**	**64**	**100**	**0.2**	**0.955**	**0.939**
128	64	100	0.2	1.000	0.922

128	64	100	0.2	0.955	0.922
128	64	100	0.2	0.955	0.911
128	64	100	0.2	1.000	0.922
128	64	100	0	0.909	0.917
128	64	100	0	1.000	0.917
128	64	100	0	1.000	0.933
128	64	100	0	0.955	0.922
128	64	100	0	1.000	0.950
128	64	50	0.6	0.864	0.900
128	64	50	0.6	0.864	0.900
128	64	50	0.6	0.682	0.872
128	64	50	0.6	0.955	0.906
128	64	50	0.6	0.955	0.906
128	64	50	0.4	0.864	0.911
128	64	50	0.4	0.955	0.928
128	64	50	0.4	0.909	0.928
128	64	50	0.4	1.000	0.906
128	64	50	0.4	0.955	0.911
128	64	50	0.2	1.000	0.922
128	64	50	0.2	0.909	0.933
128	64	50	0.2	1.000	0.950
128	64	50	0.2	0.864	0.922
128	64	50	0.2	0.864	0.939
128	64	50	0	1.000	0.906
128	64	50	0	1.000	0.922
128	64	50	0	1.000	0.906
128	64	50	0	1.000	0.900
128	64	50	0	1.000	0.939
256	128	100	0.6	0.864	0.911
256	128	100	0.6	1.000	0.906
256	128	100	0.6	0.909	0.889
256	128	100	0.6	0.864	0.911
256	128	100	0.6	0.955	0.900
256	128	100	0.4	0.955	0.906
256	128	100	0.4	1.000	0.917
256	128	100	0.4	1.000	0.917
256	128	100	0.4	1.000	0.900
256	128	100	0.4	0.909	0.911
256	128	100	0.2	1.000	0.928
256	128	100	0.2	1.000	0.917
256	128	100	0.2	0.955	0.911
256	128	100	0.2	1.000	0.911
256	128	100	0.2	0.955	0.928
256	128	100	0	1.000	0.906
256	128	100	0	0.955	0.911
256	128	100	0	1.000	0.944
256	128	100	0	0.955	0.906
256	128	100	0	1.000	0.917
256	128	50	0.6	0.864	0.906
256	128	50	0.6	0.955	0.911
256	128	50	0.6	0.909	0.900
256	128	50	0.6	0.864	0.906
256	128	50	0.6	0.864	0.900
256	128	50	0.4	0.864	0.861
256	128	50	0.4	0.909	0.911
256	128	50	0.4	0.955	0.894
256	128	50	0.4	0.909	0.911
256	128	50	0.4	0.955	0.883
256	128	50	0.2	1.000	0.906
256	128	50	0.2	0.955	0.922
256	128	50	0.2	0.955	0.917
256	128	50	0.2	1.000	0.900
256	128	50	0.2	1.000	0.917
256	128	50	0	1.000	0.917
256	128	50	0	1.000	0.950
256	128	50	0	1.000	0.944
256	128	50	0	0.955	0.922
256	128	50	0	1.000	0.922
64	32	100	0.6	1.000	0.894
64	32	100	0.6	0.682	0.872
64	32	100	0.6	0.773	0.900
64	32	100	0.6	0.818	0.906
64	32	100	0.6	0.864	0.883

64	32	100	0.4	0.864	0.917
64	32	100	0.4	0.955	0.883
64	32	100	0.4	0.773	0.894
64	32	100	0.4	0.864	0.917
64	32	100	0.4	1.000	0.917
64	32	100	0.2	0.955	0.956
64	32	100	0.2	1.000	0.922
64	32	100	0.2	1.000	0.850
64	32	100	0.2	0.909	0.928
64	32	100	0.2	0.909	0.939
64	32	100	0	1.000	0.922
64	32	100	0	0.955	0.872
64	32	100	0	1.000	0.900
64	32	100	0	1.000	0.922
64	32	100	0	1.000	0.922
64	32	50	0.6	0.864	0.861
64	32	50	0.6	0.773	0.833
64	32	50	0.6	0.727	0.833
64	32	50	0.6	0.864	0.906
64	32	50	0.6	0.909	0.861
64	32	50	0.4	1.000	0.917
64	32	50	0.4	0.818	0.867
64	32	50	0.4	0.864	0.883
64	32	50	0.4	0.727	0.900
64	32	50	0.4	0.909	0.900
64	32	50	0.2	0.909	0.922
64	32	50	0.2	0.955	0.928
64	32	50	0.2	1.000	0.944
64	32	50	0.2	0.818	0.906
64	32	50	0.2	0.955	0.911
64	32	50	0	1.000	0.939
64	32	50	0	1.000	0.894
64	32	50	0	0.955	0.889
64	32	50	0	1.000	0.911
64	32	50	0	1.000	0.889
