# Supplementary material for: moSCminer: a cell subtype classification framework based on the attention neural network integrating the single-cell multi-omics dataset on the cloud
Source: PeerJ. 2024 Feb 26;12:e17006. doi: 10.7717/peerj.17006 (PMC10903350; doi:10.7717/peerj.17006)
Supplement: Supplemental Information 2 — Grid search was adopted for the model tuning, and the hyperparameters showing the best accuracy were selected. Row with the bolded font are the hyperparmeters selected. [file peerj-12-17006-s002.pdf]

Supplementary Table S2.
Optimization results for GSE136718 dataset of the baseline methods with the different combination of the parameters.
Grid search was adopted for the model tuning, and the hyperparameters showing the best accuracy were selected.
Row with the bolded font are the hyperparmeters selected.

Support vector machine (SVM)

Kernel	Penalty parameter (C)	RBF kernel coeff (Gamma)	Accruacy
RBF	2^{-5}	2^{-15}	0.2063
RBF	2^{-5}	2^{-13}	0.2063
RBF	2^{-5}	2^{-11}	0.2063
RBF	2^{-5}	2^{-9}	0.2063
RBF	2^{-5}	2^{-7}	0.2063
RBF	2^{-5}	2^{-5}	0.2063
RBF	2^{-5}	2^{-3}	0.2063
RBF	2^{-5}	2^{-1}	0.2063
RBF	2^{-5}	2^{1}	0.2063
RBF	2^{-5}	2^{3}	0.2063
RBF	2^{-3}	2^{-15}	0.2063
RBF	2^{-3}	2^{-13}	0.2063
RBF	2^{-3}	2^{-11}	0.2063
RBF	2^{-3}	2^{-9}	0.2063
RBF	2^{-3}	2^{-7}	0.2063
RBF	2^{-3}	2^{-5}	0.3651
RBF	2^{-3}	2^{-3}	0.3333
RBF	2^{-3}	2^{-1}	0.2063
RBF	2^{-3}	2^{1}	0.2063
RBF	2^{-3}	2^{3}	0.2063
RBF	2^{-1}	2^{-15}	0.2063
RBF	2^{-1}	2^{-13}	0.2063
RBF	2^{-1}	2^{-11}	0.2063
RBF	2^{-1}	2^{-9}	0.2698
RBF	2^{-1}	2^{-7}	0.3651
RBF	2^{-1}	2^{-5}	0.7302
RBF	2^{-1}	2^{-3}	0.7937
RBF	2^{-1}	2^{-1}	0.2063
RBF	2^{-1}	2^{1}	0.2063
RBF	2^{-1}	2^{3}	0.2063
RBF	2^{-0}	2^{-15}	0.2063
RBF	2^{-0}	2^{-13}	0.2063
RBF	2^{-0}	2^{-11}	0.2063
RBF	2^{-0}	2^{-9}	0.3492
RBF	2^{-0}	2^{-7}	0.4762
RBF	2^{-0}	2^{-5}	0.8889
RBF	2^{-0}	2^{-3}	0.9206
RBF	2^{-0}	2^{-1}	0.3333
RBF	2^{-0}	2^{1}	0.2063
RBF	2^{-0}	2^{3}	0.2063
RBF	2^{1}	2^{-15}	0.2063
RBF	2^{1}	2^{-13}	0.2063
RBF	2^{1}	2^{-11}	0.2857
RBF	2^{1}	2^{-9}	0.3651
RBF	2^{1}	2^{-7}	0.7937
**RBF**	**2^{1}**	**2^{-5}**	**0.9683**
RBF	2^{1}	2^{-3}	0.9365
RBF	2^{1}	2^{-1}	0.381
RBF	2^{1}	2^{1}	0.2063
RBF	2^{1}	2^{3}	0.2063
RBF	2^{3}	2^{-15}	0.2063
RBF	2^{3}	2^{-13}	0.2857
RBF	2^{3}	2^{-11}	0.3651
RBF	2^{3}	2^{-9}	0.8095

RBF	2^{3}	2^{-7}	0.9683
RBF	2^{3}	2^{-5}	0.9524
RBF	2^{3}	2^{-3}	0.9365
RBF	2^{3}	2^{-1}	0.381
RBF	2^{3}	2^{1}	0.2063
RBF	2^{3}	2^{3}	0.2063
RBF	2^{5}	2^{-15}	0.2857
RBF	2^{5}	2^{-13}	0.3651
RBF	2^{5}	2^{-11}	0.8095
RBF	2^{5}	2^{-9}	0.9683
RBF	2^{5}	2^{-7}	0.9524
RBF	2^{5}	2^{-5}	0.9524
RBF	2^{5}	2^{-3}	0.9365
RBF	2^{5}	2^{-1}	0.381
RBF	2^{5}	2^{1}	0.2063
RBF	2^{5}	2^{3}	0.2063
Linear	2^{-5}	-	0.2063
Linear	2^{-3}	-	0.2063
Linear	2^{-1}	-	0.2063
Linear	2^{-0}	-	0.9206
Linear	2^{1}	-	0.9683
Linear	2^{3}	-	0.9683
Linear	2^{5}	-	0.9683

Random Forest (RF)

Split criteria (criterion)	# of trees (estimators)	The minimum # of samples in a leaf node (min_samples_leaf)	Accruacy
Gini impurity	100	1	0.9365
Gini impurity	100	2	0.9524
Gini impurity	100	3	0.9524
Gini impurity	100	4	0.9365
**Gini impurity**	**100**	**5**	**0.9683**
Gini impurity	300	1	0.9524
Gini impurity	300	2	0.9365
Gini impurity	300	3	0.9524
Gini impurity	300	4	0.9524
Gini impurity	300	5	0.9524
Gini impurity	500	1	0.9365
Gini impurity	500	2	0.9524
Gini impurity	500	3	0.9365
Gini impurity	500	4	0.9365
Gini impurity	500	5	0.9365
Gini impurity	700	1	0.9524
Gini impurity	700	2	0.9524
Gini impurity	700	3	0.9524
Gini impurity	700	4	0.9524
Gini impurity	700	5	0.9365
Gini impurity	900	1	0.9524
Gini impurity	900	2	0.9524
Gini impurity	900	3	0.9524
Gini impurity	900	4	0.9524
Gini impurity	900	5	0.9524
entropy	100	1	0.9365
entropy	100	2	0.9683
entropy	100	3	0.9365
entropy	100	4	0.9524
entropy	100	5	0.9365
entropy	300	1	0.9524
entropy	300	2	0.9524

entropy	300	3	0.9365
entropy	300	4	0.9524
entropy	300	5	0.9365
entropy	500	1	0.9365
entropy	500	2	0.9524
entropy	500	3	0.9365
entropy	500	4	0.9365
entropy	500	5	0.9365
entropy	700	1	0.9365
entropy	700	2	0.9524
entropy	700	3	0.9524
entropy	700	4	0.9365
entropy	700	5	0.9365
entropy	900	1	0.9365
entropy	900	2	0.9365
entropy	900	3	0.9524
entropy	900	4	0.9524
entropy	900	5	0.9365

Logistic Regression (LR)

max_iter (maximum number of iterations to converge)	Penalty parameter (C)	Accuracy
100	0.03125	0.6667
100	0.125	0.8571
**100**	**0.5**	**0.9683**
100	1	0.9683
100	2	0.9524
100	8	0.9524
100	32	0.9524
200	0.03125	0.6667
200	0.125	0.8571
200	0.5	0.9683
200	1	0.9683
200	2	0.9524
200	8	0.9524
200	32	0.9524
300	0.03125	0.6667
300	0.125	0.8571
300	0.5	0.9683
300	1	0.9683
300	2	0.9524
300	8	0.9524
300	32	0.9524
400	0.03125	0.6667
400	0.125	0.8571
400	0.5	0.9683
400	1	0.9683
400	2	0.9524
400	8	0.9524
400	32	0.9524
500	0.03125	0.6667
500	0.125	0.8571
500	0.5	0.9683
500	1	0.9683
500	2	0.9524
500	8	0.9524
500	32	0.9524
