# Supplementary material for: moSCminer: a cell subtype classification framework based on the attention neural network integrating the single-cell multi-omics dataset on the cloud
Source: PeerJ. 2024 Feb 26;12:e17006. doi: 10.7717/peerj.17006 (PMC10903350; doi:10.7717/peerj.17006)
Supplement: Supplemental Information 3 — Grid search was adopted for the model tuning, and the hyperparameters showing the best accuracy were selected. Row with the bolded font are the hyperparmeters selected. [file peerj-12-17006-s003.pdf]

Supplementary Table S3.
Optimization results for GSE154762 dataset of the baseline methods with the different combination of the parameters.
Grid search was adopted for the model tuning, and the hyperparameters showing the best accuracy were selected.
Row with the bolded font are the hyperparmeters selected.

Support vector machine (SVM)

Kernel	Penalty parameter (C)	RBF kernel coeff (Gamma)	Accruacy
RBF	2^{-5}	2^{-15}	0.3000
RBF	2^{-5}	2^{-13}	0.3000
RBF	2^{-5}	2^{-11}	0.3000
RBF	2^{-5}	2^{-9}	0.3000
RBF	2^{-5}	2^{-7}	0.2963
RBF	2^{-5}	2^{-5}	0.4667
RBF	2^{-5}	2^{-3}	0.4593
RBF	2^{-5}	2^{-1}	0.2148
RBF	2^{-5}	2^{1}	0.1889
RBF	2^{-5}	2^{3}	0.2630
RBF	2^{-3}	2^{-15}	0.3000
RBF	2^{-3}	2^{-13}	0.3000
RBF	2^{-3}	2^{-11}	0.3000
RBF	2^{-3}	2^{-9}	0.3000
RBF	2^{-3}	2^{-7}	0.5111
RBF	2^{-3}	2^{-5}	0.6000
RBF	2^{-3}	2^{-3}	0.5333
RBF	2^{-3}	2^{-1}	0.2556
RBF	2^{-3}	2^{1}	0.1889
RBF	2^{-3}	2^{3}	0.2630
RBF	2^{-1}	2^{-15}	0.3000
RBF	2^{-1}	2^{-13}	0.3000
RBF	2^{-1}	2^{-11}	0.3000
RBF	2^{-1}	2^{-9}	0.5074
RBF	2^{-1}	2^{-7}	0.6667
RBF	2^{-1}	2^{-5}	0.7148
RBF	2^{-1}	2^{-3}	0.7148
RBF	2^{-1}	2^{-1}	0.3778
RBF	2^{-1}	2^{1}	0.1889
RBF	2^{-1}	2^{3}	0.2630
RBF	2^{-0}	2^{-15}	0.3000
RBF	2^{-0}	2^{-13}	0.3000
RBF	2^{-0}	2^{-11}	0.4333
RBF	2^{-0}	2^{-9}	0.5778
RBF	2^{-0}	2^{-7}	0.7000
RBF	2^{-0}	2^{-5}	0.7407
RBF	2^{-0}	2^{-3}	0.8333
RBF	2^{-0}	2^{-1}	0.4667
RBF	2^{-0}	2^{1}	0.2111
RBF	2^{-0}	2^{3}	0.1963
RBF	2^{1}	2^{-15}	0.3000
RBF	2^{1}	2^{-13}	0.3000
RBF	2^{1}	2^{-11}	0.5074
RBF	2^{1}	2^{-9}	0.6778
RBF	2^{1}	2^{-7}	0.7148
RBF	2^{1}	2^{-5}	0.8185
RBF	2^{1}	2^{-3}	0.8407
RBF	2^{1}	2^{-1}	0.5037
RBF	2^{1}	2^{1}	0.2074
RBF	2^{1}	2^{3}	0.1815
RBF	2^{3}	2^{-15}	0.3000
RBF	2^{3}	2^{-13}	0.5111
RBF	2^{3}	2^{-11}	0.6815
RBF	2^{3}	2^{-9}	0.7148

RBF	2^{3}	2^{-7}	0.8185
RBF	2^{3}	2^{-5}	0.8481
RBF	2^{3}	2^{-3}	0.8407
RBF	2^{3}	2^{-1}	0.5037
RBF	2^{3}	2^{1}	0.2074
RBF	2^{3}	2^{3}	0.1815
RBF	2^{5}	2^{-15}	0.5111
RBF	2^{5}	2^{-13}	0.6815
RBF	2^{5}	2^{-11}	0.7185
RBF	2^{5}	2^{-9}	0.8074
RBF	2^{5}	2^{-7}	0.8481
**RBF**	**2^{5}**	**2^{-5}**	**0.8593**
RBF	2^{5}	2^{-3}	0.8407
RBF	2^{5}	2^{-1}	0.5037
RBF	2^{5}	2^{1}	0.2074
RBF	2^{5}	2^{3}	0.1815
Linear	2^{-5}	-	0.4667
Linear	2^{-3}	-	0.6000
Linear	2^{-1}	-	0.7148
Linear	2^{-0}	-	0.8333
Linear	2^{1}	-	0.8407
Linear	2^{3}	-	0.8481
Linear	2^{5}	-	0.8593

Random Forest (RF)

Split criteria (criterion)	# of trees (estimators)	The minimum # of samples in a leaf node (min_samples_leaf)	Accruacy
Gini impurity	100	1	0.9111
Gini impurity	100	2	0.9000
Gini impurity	100	3	0.8926
Gini impurity	100	4	0.8926
Gini impurity	100	5	0.8963
Gini impurity	300	1	0.8963
Gini impurity	300	2	0.9185
Gini impurity	300	3	0.9074
Gini impurity	300	4	0.8889
Gini impurity	300	5	0.8889
Gini impurity	500	1	0.9074
Gini impurity	500	2	0.9037
Gini impurity	500	3	0.9111
Gini impurity	500	4	0.9000
Gini impurity	500	5	0.9000
Gini impurity	700	1	0.9185
Gini impurity	700	2	0.8963
Gini impurity	700	3	0.9037
Gini impurity	700	4	0.9000
Gini impurity	700	5	0.8963
Gini impurity	900	1	0.9074
Gini impurity	900	2	0.9000
Gini impurity	900	3	0.9148
Gini impurity	900	4	0.9074
Gini impurity	900	5	0.8889
entropy	100	1	0.9111
entropy	100	2	0.8926
entropy	100	3	0.9111
entropy	100	4	0.8852
entropy	100	5	0.8852
entropy	300	1	0.9185
entropy	300	2	0.9111

**entropy**	**300**	**3**	**0.9259**
entropy	300	4	0.8926
entropy	300	5	0.9037
entropy	500	1	0.9111
entropy	500	2	0.9259
entropy	500	3	0.9074
entropy	500	4	0.9037
entropy	500	5	0.9074
entropy	700	1	0.8963
entropy	700	2	0.9000
entropy	700	3	0.9074
entropy	700	4	0.9074
entropy	700	5	0.8963
entropy	900	1	0.9074
entropy	900	2	0.9037
entropy	900	3	0.9037
entropy	900	4	0.9074
entropy	900	5	0.9000

Logistic Regression (LR)

max_iter (maximum number of iterations to converge)	Penalty parameter (C)	Accuracy
100	0.03125	0.7185
100	0.125	0.7407
100	0.5	0.7963
100	1	0.7963
100	2	0.8222
**100**	**8**	**0.8259**
100	32	0.8222
200	0.03125	0.7185
200	0.125	0.7407
200	0.5	0.7963
200	1	0.7963
200	2	0.8222
200	8	0.8259
200	32	0.8222
300	0.03125	0.7185
300	0.125	0.7407
300	0.5	0.7963
300	1	0.7963
300	2	0.8222
300	8	0.8259
300	32	0.8222
400	0.03125	0.7185
400	0.125	0.7407
400	0.5	0.7963
400	1	0.7963
400	2	0.8222
400	8	0.8259
400	32	0.8222
500	0.03125	0.7185
500	0.125	0.7407
500	0.5	0.7963
500	1	0.7963
500	2	0.8222
500	8	0.8259
500	32	0.8222
