# Supplementary material for: moSCminer: a cell subtype classification framework based on the attention neural network integrating the single-cell multi-omics dataset on the cloud
Source: PeerJ. 2024 Feb 26;12:e17006. doi: 10.7717/peerj.17006 (PMC10903350; doi:10.7717/peerj.17006)
Supplement: Supplemental Information 4 — Grid search was adopted for the model tuning, and the hyperparameters showing the best accuracy were selected. Row with the bolded font are the hyperparmeters selected. [file peerj-12-17006-s004.pdf]

Supplementary Table S4.
Optimization results for GSE140203 dataset of the baseline methods with the different combination of the parameters.
Grid search was adopted for the model tuning, and the hyperparameters showing the best accuracy were selected.
Row with the bolded font are the hyperparmeters selected.

Support vector machine (SVM)

Kernel	Penalty parameter (C)	RBF kernel coeff (Gamma)	Accruacy
RBF	2^{-5}	2^{-15}	0.2431
RBF	2^{-5}	2^{-13}	0.2431
RBF	2^{-5}	2^{-11}	0.2431
RBF	2^{-5}	2^{-9}	0.2431
RBF	2^{-5}	2^{-7}	0.3844
RBF	2^{-5}	2^{-5}	0.5282
RBF	2^{-5}	2^{-3}	0.2525
RBF	2^{-5}	2^{-1}	0.2431
RBF	2^{-5}	2^{1}	0.2431
RBF	2^{-5}	2^{3}	0.2431
RBF	2^{-3}	2^{-15}	0.2431
RBF	2^{-3}	2^{-13}	0.2431
RBF	2^{-3}	2^{-11}	0.2432
RBF	2^{-3}	2^{-9}	0.4210
RBF	2^{-3}	2^{-7}	0.6439
RBF	2^{-3}	2^{-5}	0.7011
RBF	2^{-3}	2^{-3}	0.4982
RBF	2^{-3}	2^{-1}	0.2431
RBF	2^{-3}	2^{1}	0.2431
RBF	2^{-3}	2^{3}	0.2431
RBF	2^{-1}	2^{-15}	0.2431
RBF	2^{-1}	2^{-13}	0.2432
RBF	2^{-1}	2^{-11}	0.4297
RBF	2^{-1}	2^{-9}	0.6569
RBF	2^{-1}	2^{-7}	0.7448
RBF	2^{-1}	2^{-5}	0.7742
RBF	2^{-1}	2^{-3}	0.7006
RBF	2^{-1}	2^{-1}	0.2431
RBF	2^{-1}	2^{1}	0.2431
RBF	2^{-1}	2^{3}	0.2431
RBF	2^{-0}	2^{-15}	0.2431
RBF	2^{-0}	2^{-13}	0.3085
RBF	2^{-0}	2^{-11}	0.5730
RBF	2^{-0}	2^{-9}	0.7082
RBF	2^{-0}	2^{-7}	0.7732
RBF	2^{-0}	2^{-5}	0.7928
RBF	2^{-0}	2^{-3}	0.7452
RBF	2^{-0}	2^{-1}	0.2431
RBF	2^{-0}	2^{1}	0.2431
RBF	2^{-0}	2^{3}	0.2431
RBF	2^{1}	2^{-15}	0.2432
RBF	2^{1}	2^{-13}	0.4321
RBF	2^{1}	2^{-11}	0.6594
RBF	2^{1}	2^{-9}	0.7509
RBF	2^{1}	2^{-7}	0.7877
RBF	2^{1}	2^{-5}	0.7973
RBF	2^{1}	2^{-3}	0.7571
RBF	2^{1}	2^{-1}	0.2431
RBF	2^{1}	2^{1}	0.2431
RBF	2^{1}	2^{3}	0.2431
RBF	2^{3}	2^{-15}	0.4334
RBF	2^{3}	2^{-13}	0.6600
RBF	2^{3}	2^{-11}	0.7506
RBF	2^{3}	2^{-9}	0.7877

RBF	2^{3}	2^{-7}	0.7973
**RBF**	**2^{3}**	**2^{-5}**	**0.8030**
RBF	2^{3}	2^{-3}	0.7569
RBF	2^{3}	2^{-1}	0.2431
RBF	2^{3}	2^{1}	0.2431
RBF	2^{3}	2^{3}	0.2431
RBF	2^{5}	2^{-15}	0.6602
RBF	2^{5}	2^{-13}	0.7514
RBF	2^{5}	2^{-11}	0.7861
RBF	2^{5}	2^{-9}	0.7970
RBF	2^{5}	2^{-7}	0.8022
RBF	2^{5}	2^{-5}	0.8033
RBF	2^{5}	2^{-3}	0.7569
RBF	2^{5}	2^{-1}	0.2431
RBF	2^{5}	2^{1}	0.2431
RBF	2^{5}	2^{3}	0.2431
Linear	2^{-5}	-	0.7866
Linear	2^{-3}	-	0.7949
Linear	2^{-1}	-	0.7897
Linear	2^{-0}	-	0.7832
Linear	2^{1}	-	0.7881
Linear	2^{3}	-	0.7864
Linear	2^{5}	-	0.7814

Random Forest (RF)

Split criteria (criterion)	# of trees (estimators)	The minimum # of samples in a leaf node (min_samples_leaf)	Accruacy
Gini impurity	100	1	0.9234
Gini impurity	100	2	0.9265
Gini impurity	100	3	0.9232
Gini impurity	100	4	0.9248
Gini impurity	100	5	0.9198
Gini impurity	300	1	0.9322
Gini impurity	300	2	0.9308
Gini impurity	300	3	0.9349
Gini impurity	300	4	0.9305
Gini impurity	300	5	0.9269
Gini impurity	500	1	0.9339
Gini impurity	500	2	0.9364
Gini impurity	500	3	0.9330
Gini impurity	500	4	0.9327
Gini impurity	500	5	0.9305
Gini impurity	700	1	0.9390
Gini impurity	700	2	0.9375
Gini impurity	700	3	0.9353
Gini impurity	700	4	0.9321
Gini impurity	700	5	0.9322
Gini impurity	900	1	0.9395
Gini impurity	900	2	0.9366
Gini impurity	900	3	0.9342
Gini impurity	900	4	0.9349
Gini impurity	900	5	0.9307
entropy	100	1	0.9313
entropy	100	2	0.9300
entropy	100	3	0.9310
entropy	100	4	0.9330
entropy	100	5	0.9290
entropy	300	1	0.9395
entropy	300	2	0.9378

entropy	300	3	0.9395
entropy	300	4	0.9358
entropy	300	5	0.9386
entropy	500	1	0.9420
entropy	500	2	0.9428
entropy	500	3	0.9411
entropy	500	4	0.9394
entropy	500	5	0.9401
entropy	700	1	0.9437
**entropy**	**700**	**2**	**0.9442**
entropy	700	3	0.9411
entropy	700	4	0.9414
entropy	700	5	0.9411
entropy	900	1	0.9432
entropy	900	2	0.9421
entropy	900	3	0.9429
entropy	900	4	0.9415
entropy	900	5	0.9397

Logistic Regression (LR)

max_iter (maximum number of iterations to converge)	Penalty parameter (C)	Accuracy
100	0.03125	0.7639
100	0.125	0.7886
100	0.5	0.8069
100	1	0.8148
100	2	0.8215
**100**	**8**	**0.8226**
100	32	0.8176
200	0.03125	0.7639
200	0.125	0.7886
200	0.5	0.8069
200	1	0.8148
200	2	0.8215
200	8	0.8226
200	32	0.8176
300	0.03125	0.7639
300	0.125	0.7886
300	0.5	0.8069
300	1	0.8148
300	2	0.8215
300	8	0.8226
300	32	0.8176
400	0.03125	0.7639
400	0.125	0.7886
400	0.5	0.8069
400	1	0.8148
400	2	0.8215
400	8	0.8226
400	32	0.8176
500	0.03125	0.7639
500	0.125	0.7886
500	0.5	0.8069
500	1	0.8148
500	2	0.8215
500	8	0.8226
500	32	0.8176
