# Supplementary material for: moSCminer: a cell subtype classification framework based on the attention neural network integrating the single-cell multi-omics dataset on the cloud
Source: PeerJ. 2024 Feb 26;12:e17006. doi: 10.7717/peerj.17006 (PMC10903350; doi:10.7717/peerj.17006)
Supplement: Supplemental Information 5 [file peerj-12-17006-s005.pdf]

SupplementaryMaterial S5.
Average cell subtype-wise performance results for cell subtype predictions of moSCminer with its variant and the baseline methods based on 5-fold cross-validation.

GSE136718	Cell subtype	moSCminer (omics-attn)	moSCminer (no-attn)	RF	SVM	LR	NB
Accuracy	2cell	**1.000**	**1.000**	**1.000**	**1.000**	**1.000**	0.971
	4cell	**0.991**	0.962	0.971	0.976	0.971	0.924
	8cell	0.995	0.995	**1.000**	0.995	0.991	0.910
	ICM	**1.000**	**1.000**	**1.000**	**1.000**	0.995	0.929
	Late4cell	**0.991**	0.962	0.971	0.971	0.967	0.933
	Morula	0.995	0.995	**1.000**	**1.000**	0.995	0.943
	TE	**1.000**	**1.000**	**1.000**	**1.000**	0.995	0.924
	Zygote	**1.000**	**1.000**	**1.000**	**1.000**	**1.000**	0.981
F1-score	2cell	**1.000**	**1.000**	**1.000**	**1.000**	**1.000**	0.870
	4cell	**0.977**	0.906	0.930	0.937	0.928	0.808
	8cell	0.960	0.987	**1.000**	0.985	0.973	0.736
	ICM	**1.000**	**1.000**	**1.000**	**1.000**	0.987	0.784
	Late4cell	**0.953**	0.780	0.847	0.825	0.798	0.565
	Morula	0.982	0.978	**1.000**	**1.000**	0.978	0.671
	TE	**1.000**	**1.000**	**1.000**	**1.000**	0.978	0.576
	Zygote	**1.000**	**1.000**	**1.000**	**1.000**	**1.000**	0.820
ROC-AUC	2cell	**1.000**	**1.000**	**1.000**	**1.000**	**1.000**	0.967
	4cell	**0.986**	0.952	0.982	0.985	0.982	0.921
	8cell	0.967	0.988	**1.000**	0.997	0.994	0.900
	ICM	**1.000**	**1.000**	**1.000**	**1.000**	0.997	0.869
	Late4cell	**0.981**	0.894	0.879	0.858	0.842	0.760
	Morula	0.997	0.997	**1.000**	**1.000**	0.980	0.789
	TE	**1.000**	**1.000**	**1.000**	**1.000**	0.980	0.819
	Zygote	**1.000**	**1.000**	**1.000**	**1.000**	**1.000**	0.867

GSE154762	Cell subtype	moSCminer (omics-attn)	moSCminer (no-attn)	RF	SVM	LR	NB
Accuracy	FGO	**0.993**	0.984	0.992	0.989	0.984	0.922
	GO1	**0.994**	0.971	0.986	0.983	0.972	0.940
	GO2	**0.992**	0.971	0.983	0.982	0.971	0.950
	Granulosa	0.991	0.984	0.990	**0.994**	0.993	0.912
	Immune	0.997	0.999	0.999	**1.000**	**1.000**	0.980
	MI	**0.982**	0.945	0.976	0.961	0.949	0.859
	MII	**0.989**	0.954	0.981	0.971	0.963	0.891
	StromaC1	**0.998**	0.912	0.953	0.920	0.911	0.797
	StromaC2	**0.993**	0.911	0.954	0.923	0.909	0.786
F1-score	FGO	**0.953**	0.911	0.965	0.932	0.907	0.560
	GO1	**0.922**	0.712	0.861	0.829	0.741	0.537
	GO2	**0.870**	0.600	0.741	0.740	0.607	0.504
	Granulosa	0.954	0.910	0.941	**0.973**	0.963	0.613
	Immune	0.978	0.978	0.921	**1.000**	**1.000**	0.160
	MI	**0.950**	0.844	0.928	0.884	0.854	0.544
	MII	**0.942**	0.768	0.898	0.848	0.808	0.505
	StromaC1	**0.995**	0.790	0.890	0.808	0.784	0.468
	StromaC2	**0.982**	0.780	0.882	0.810	0.781	0.457
ROC-AUC	FGO	**0.986**	0.944	0.961	0.949	0.944	0.763
	GO1	**0.981**	0.913	0.965	0.946	0.919	0.874
	GO2	**0.946**	0.754	0.811	0.840	0.760	0.765
	Granulosa	0.956	0.926	0.980	**0.991**	0.968	0.818
	Immune	0.999	0.999	0.930	**1.000**	**1.000**	0.550
	MI	**0.984**	0.914	0.963	0.936	0.920	0.715
	MII	**0.951**	0.873	0.933	0.914	0.885	0.744
	StromaC1	**0.999**	0.872	0.936	0.882	0.865	0.661
	StromaC2	**0.992**	0.859	0.918	0.877	0.863	0.661

GSE140203	Cell subtype	moSCminer (omics-attn)	moSCminer (no-attn)	RF	SVM	LR	NB
Accuracy	Basal	**0.998**	0.966	0.994	0.927	0.953	0.802
	Dermal Fibroblast	**0.999**	0.994	0.997	0.993	0.995	0.971
	Dermal Papilla	**0.998**	0.995	0.996	0.995	0.995	0.969
	Dermal Sheath	**0.999**	0.997	0.996	0.996	0.996	0.966
	Endothelial	**0.999**	0.994	0.998	0.993	0.993	0.976
	Granular	**0.999**	0.993	0.995	0.992	0.992	0.970
	Hair Shaft-cuticle-cortex	**0.998**	0.987	0.997	0.983	0.986	0.972
	IRS	**0.999**	0.989	0.997	0.985	0.987	0.969
	Infundibulum	**0.997**	0.964	0.984	0.947	0.958	0.887
	Isthmus	**0.999**	0.991	0.998	0.986	0.989	0.919
	K6+ Bulge Companion Layer	**0.999**	0.994	0.997	0.991	0.993	0.982
	Macrophage DC	**1.000**	0.997	0.998	0.995	0.996	0.982
	Medulla	**0.998**	0.985	0.996	0.982	0.984	0.964
	Melanocyte	**1.000**	0.999	0.998	0.999	0.999	0.989
	ORS	**0.999**	0.993	0.998	0.992	0.992	0.979
	Schwann Cell	**0.999**	0.998	0.999	0.996	0.997	0.852
	Sebaceous Gland	**1.000**	0.999	0.999	0.998	0.998	0.978
	Spinous	**0.997**	0.971	0.994	0.955	0.966	0.910
	TAC-1	**0.995**	0.959	0.972	0.944	0.952	0.908
	TAC-2	**0.998**	0.981	0.995	0.979	0.980	0.969
	ahighCD34+ bulge	**0.999**	0.986	0.998	0.978	0.983	0.906
	alowCD34+ bulge	**0.998**	0.981	0.997	0.975	0.978	0.943
F1-score	Basal	**0.995**	0.929	0.987	0.858	0.905	0.651
	Dermal Fibroblast	**0.980**	0.918	0.955	0.898	0.922	0.286
	Dermal Papilla	**0.968**	0.884	0.909	0.884	0.892	0.281
	Dermal Sheath	**0.945**	0.873	0.804	0.834	0.845	0.126
	Endothelial	**0.976**	0.901	0.958	0.869	0.877	0.330
	Granular	**0.924**	0.503	0.576	0.373	0.391	0.105
	Hair Shaft-cuticle-cortex	**0.978**	0.822	0.953	0.769	0.806	0.578
	IRS	**0.969**	0.726	0.909	0.635	0.676	0.397
	Infundibulum	**0.987**	0.865	0.941	0.801	0.841	0.330
	Isthmus	**0.979**	0.775	0.941	0.629	0.716	0.228
	K6+ Bulge Companion Layer	**0.969**	0.811	0.903	0.689	0.763	0.273
	Macrophage DC	**0.977**	0.805	0.828	0.625	0.728	0.075
	Medulla	**0.967**	0.755	0.930	0.671	0.718	0.162
	Melanocyte	**0.961**	0.900	0.837	0.884	0.873	0.378
	ORS	**0.982**	0.896	0.959	0.877	0.878	0.539
	Schwann Cell	**0.941**	0.724	0.823	0.456	0.542	0.035
	Sebaceous Gland	**0.992**	0.887	0.847	0.839	0.851	0.273
	Spinous	**0.987**	0.853	0.972	0.763	0.823	0.195
	TAC-1	**0.978**	0.804	0.881	0.742	0.777	0.285
	TAC-2	**0.965**	0.685	0.919	0.615	0.648	0.283
	ahighCD34+ bulge	**0.991**	0.853	0.973	0.767	0.814	0.449
	alowCD34+ bulge	**0.985**	0.836	0.973	0.788	0.811	0.107
ROC-AUC	Basal	**0.996**	0.955	0.989	0.919	0.946	0.789
	Dermal Fibroblast	**0.988**	0.971	0.985	0.950	0.959	0.585
	Dermal Papilla	**0.983**	0.928	0.968	0.930	0.930	0.616
	Dermal Sheath	**0.965**	0.919	0.837	0.890	0.896	0.570
	Endothelial	**0.990**	0.946	0.974	0.917	0.933	0.602
	Granular	**0.967**	0.698	0.703	0.629	0.648	0.585
	Hair Shaft-cuticle-cortex	**0.988**	0.909	0.955	0.879	0.897	0.760
	IRS	**0.982**	0.860	0.917	0.809	0.818	0.736
	Infundibulum	**0.994**	0.934	0.987	0.900	0.920	0.602
	Isthmus	**0.984**	0.876	0.944	0.785	0.830	0.736
	K6+ Bulge Companion Layer	**0.975**	0.910	0.912	0.812	0.863	0.607
	Macrophage DC	**0.978**	0.865	0.855	0.747	0.830	0.570
	Medulla	**0.983**	0.867	0.950	0.804	0.842	0.552
	Melanocyte	**0.967**	0.927	0.861	0.902	0.907	0.784
	ORS	**0.988**	0.945	0.961	0.925	0.933	0.694
	Schwann Cell	**0.953**	0.806	0.853	0.658	0.712	0.665
	Sebaceous Gland	**0.992**	0.937	0.868	0.888	0.894	0.818

	Spinous	**0.994**	0.918	0.990	0.861	0.902	0.554
	TAC-1	**0.992**	0.893	0.982	0.871	0.886	0.586
	TAC-2	**0.980**	0.824	0.925	0.770	0.795	0.597
	ahighCD34+ bulge	**0.993**	0.927	0.974	0.868	0.893	0.844
	alowCD34+ bulge	**0.994**	0.916	0.978	0.888	0.906	0.529
