# Supplementary material for: moSCminer: a cell subtype classification framework based on the attention neural network integrating the single-cell multi-omics dataset on the cloud
Source: PeerJ. 2024 Feb 26;12:e17006. doi: 10.7717/peerj.17006 (PMC10903350; doi:10.7717/peerj.17006)
Supplement: Supplemental Information 6 [file peerj-12-17006-s006.pdf]

SupplementaryMaterial S6.

Top 30 features showing the highest average attention scores from each omics, related to the subtype classification within cells.

GSE136718

Gene expression	DNA methylation	DNA accessibility
Prdm10	Sfi1_cpg	Ncam1_acc
A530040E14Rik	RP23-191E3.2_cpg	Zim1_acc
Wipf1	H2-Q1_cpg	RP23-282N17.2_acc
AC123856.4-3	Etl4_cpg	Sema4a_acc
Bptf	Kalrn_cpg	Ankrd24_acc
H2-Q1	Ndrg4_cpg	Trim2_acc
Ranbp3	Fkbp5_cpg	Phactr3_acc
Mcart1	Pcdha5_cpg	H2-Q1_acc
8430427H17Rik	Itsn1_cpg	SNORA17_acc
Ndst1	Triobp_cpg	Kcng1_acc
2410001C21Rik	Sp110_cpg	Shank1_acc
AC116997.3-1	Col23a1_cpg	Brunol5_acc
Fkbp5	Tmem116_cpg	Kpna1_acc
2410025L10Rik	Trim2_cpg	C130026I21Rik_acc
RP23-326E2.2	Fhad1_cpg	Pde4d_acc
Kalrn	Ncam1_cpg	Ccdc64_acc
Furin	Smarcd1_cpg	9330129D05Rik_acc
Tmem194	Kpna1_cpg	Mcart1_acc
Etl4	AC123856.4-3_cpg	Shb_acc
Prkcd	C130026I21Rik_cpg	Etfb_acc
C130026I21Rik	Kcng1_cpg	AC123856.4-3_acc
Taf3	Synj2_cpg	Sh3bp5_acc
4933407C03Rik	Ccdc64_cpg	Capns1_acc
Tef	Ank3_cpg	Tspan5_acc
Itsn1	Wipf1_cpg	4933407C03Rik_acc
Ccdc64	Sh3bp5_cpg	Elfn1_acc
Sp110	Coro2a_cpg	Prdm10_acc
Sp140	4933407C03Rik_cpg	2410001C21Rik_acc
Cic	9330129D05Rik_cpg	Pcdha5_acc
Fam129b	Shank1_cpg	Ntm_acc

GSE154762

Gene expression	DNA methylation	DNA accessibility
RORA	C1RL_cpg	SOX6_acc
ABCA12	PCDH7_cpg	ARHGAP15_acc
RAB11A	SGCA_cpg	CTNNA3_acc
C1orf21	MEPCE_cpg	FAF1_acc
TJP1	LINC00484_cpg	FRMD6_acc
COBLL1	NBPF1_cpg	IFT43_acc
RBMS1	ANKRD18CP_cpg	WDR64_acc

SLC35F2	ENPP6_cpg	LRP1B_acc
SCP2	GATA5_cpg	TMEFF2_acc
SETBP1	FANK1_cpg	PAPPA2_acc
ANK3	BTN2A2_cpg	ERC1_acc
CAMK1D	C19orf25_cpg	SOX9-AS1_acc
TSHZ2	NR2F1_cpg	RNF219-AS1_acc
MAP2	ERI2_cpg	VIT_acc
CYFIP1	MIR570_cpg	HERC2P3_acc
ADD3	TEKT4P2_cpg	ATAD2B_acc
MAPK8	ZNF595_cpg	AK7_acc
SPTBN1	MST1P2_cpg	ADAMTS18_acc
GAS7	ANKRD30BP2_cpg	LYST_acc
TMOD3	LINC00960_cpg	PPP4R4_acc
MYO5B	LINC00491_cpg	MAP4K3_acc
MREG	LINC00273_cpg	SUSD4_acc
FOXN3	ROCK1P1_cpg	STON1-GTF2A1L_acc
CERS6	FEZF1-AS1_cpg	RPH3A_acc
NEBL	DLEU1_cpg	SMG6_acc
PARD3	LRFN5_cpg	SLCO3A1_acc
CCNY	NME8_cpg	BMPR1A_acc
KAZN	NETO1_cpg	EPC1_acc
JMJD1C	MUC16_cpg	MYO3B_acc
FRMD6	C8orf58_cpg	DCLK1_acc
