# Supplementary material for: moSCminer: a cell subtype classification framework based on the attention neural network integrating the single-cell multi-omics dataset on the cloud
Source: PeerJ. 2024 Feb 26;12:e17006. doi: 10.7717/peerj.17006 (PMC10903350; doi:10.7717/peerj.17006)
Supplement: Supplemental Information 7 — Gene expression and DNA accessibility were denoted as ‘gene’ and ‘acc’, respectively. [file peerj-12-17006-s007.pdf]

SupplementaryMaterial S7.

Average cell subtype classification performance using different omics profiles in GSE140203.
Gene expression and DNA accessibility were denoted as ‘gene’ and ‘acc’, respectively.

# of omics	Metric	moSCminer	RF	SVM	LR	NB
Multi-omics	Accuracy	**0.983**	0.944	0.790	0.829	0.382
	F1-score	**0.983**	0.944	0.786	0.827	0.379
Single omics (gene)	Accuracy	**0.768**	0.640	0.759	0.753	0.179
	F1-score	**0.760**	0.604	0.754	0.749	0.246
Single omics (acc)	Accuracy	**0.950**	0.941	0.589	0.762	0.287
	F1-score	**0.951**	0.941	0.525	0.754	0.256
